# Expanding treatment pathways for sexually abusive behaviour in young people: an examination of Therapeutic Treatment Orders

**DOI:** 10.1080/13218719.2023.2243303

**Published:** 2024-01-08

**Authors:** Erika Fortunato, Nina Papalia, James R. P. Ogloff

**Affiliations:** Centre for Forensic Behavioural Science, Swinburne University of Technology and Victorian Institute of Forensic Mental Health, Alphington, VIC, Australia

**Keywords:** diversion, mandatory treatment, problem sexual behaviour, sexual abuse, sexually abusive behaviour, sexual offending, Therapeutic Treatment Order, treatment pathways, youth justice, youth offending

## Abstract

Young people who engage in sexually abusive behaviour account for a substantial number of sexual offences worldwide. Despite this, a limited body of work has explored the optimal pathways into treatment for these young people. This is an important question to explore given the iatrogenic effects of receiving treatment following incarceration and burgeoning legislative frameworks focusing on the diversion of youth who sexually offend. In Victoria, Australia, Therapeutic Treatment Orders were introduced to mandate young people with sexually abusive behaviours to community-based treatment without undergoing formal criminal justice processes. It is possible this unique treatment pathway helps overcome several limitations associated with traditional pathways. This article reviews existing research on pathways to treatment for young people who engage in sexually abusive behaviour before detailing Therapeutic Treatment Orders, and the role they may play in unionising criminal justice and diversion treatment frameworks. Considerations for future research are also explored.

Young people aged from 10 to 17 years are responsible for a significant proportion of sexual abuse incidents, especially against children and younger siblings (Malvaso et al., 2020; Smallbone & Rayment‐Mchugh, 2013). Between 2020 and 2021, young people aged from 10 to 18 years accounted for around 16% of ‘sexual assault and related offences’ in Australia (Australian Bureau of Statistics, 2022). Global estimates are even higher, with between 20% and 50% of sexual offences perpetrated by people aged below 18 years in countries such as the United States (Federal Bureau of Investigation, 2019; Prinsloo & da Costa, 2017). The large incidence of young people who engage in sexually abusive behaviour (Y-SAB) highlights that the management and treatment of these young people is a matter of international importance.

A number of adverse outcomes have been associated with Y-SAB. Firstly, although most young people with sexually abusive behaviour do not sexually reoffend, a substantial number continue sexual and general reoffending into adulthood (Beaudry-Cyr et al., 2017; Reale et al., 2020). Secondly, victim-survivors of these harmful behaviours can experience significant mental health and emotional difficulties akin to having been offended against by an adult (Royal Commission, 2017; Shaw et al., 2000). Thus, there is a need to continue delineating the treatment strategies that most successfully prevent acts of sexual violence perpetrated by young people.

Due to their unique developmental needs, there has been a shift in focus internationally from punishment-oriented legal policies to early intervention and rehabilitation for Y-SAB as well as young people who perpetrate non-sexual offences (Y-NSO; Brownlie, 2003; McAlinden, 2011). Specifically, diversion is an increasingly utilised legal provision for managing young people who engage in offending behaviours. Diversion helps to ensure young people access treatments that address their offending behaviour while not exposing them to the potentially adverse effects of a formal criminal justice response (Zimring, 2000). For Y-SAB in particular, diversion pathways have been adopted in countries including the United Kingdom (Haines et al., 2013; Smith et al., 2014) and South Africa (Gxubane, 2016; Steyn, 2012). In Victoria, Australia, the governing legislature introduced the *Children, Youth & Families Act 2005* (Vic) *(CYFA)* with the provision of Therapeutic Treatment Orders (TTOs). TTOs are legal orders made by the Children’s Court of Victoria which mandate Y-SAB aged between 10 and 18 years to undertake community-based treatment. If young people successfully engage in and complete treatment, official criminal action is not pursued against them. Thus, TTOs act as a diversion pathway.

Despite burgeoning legislative frameworks, there is limited empirical research on the efficacy of diversion pathways for Y-SAB. As Van der Merwe and Dawes (2009) highlight, this is problematic given that diverting young offenders to treatments that may not be appropriately addressing their offending is ethically and legally precarious. To address this concern, Souverein et al. (2019) emphasised the necessity of international collaboration to determine ‘what works’ for young people who have engaged in offending behaviours. This includes looking to global policies and practices in search of the most effective means of targeting youth offending.

Building on this background, this article seeks to add to the international literature by describing a diversion pathway for Y-SAB implemented in Victoria, Australia. The article gives particular focus to diversion programmes that provide young people with access to treatment for sexually abusive behaviours, effectively focusing on diversion as a treatment pathway. We begin by providing a brief overview of Y-SAB, including common individual and offending characteristics, before discussing the utility of court mandated treatment following adjudication. We then consider the strengths and limitations of utilising diversionary pathways into treatment for young people. Next, we provide a critical overview of existing research on diversion pathways for Y-SAB before focusing on the treatment service model in Victoria, Australia, including a critical analysis of TTOs. We ultimately contend that although TTOs (and, arguably, other diversionary treatment pathways) are a potentially promising avenue that may remedy some of the negatives of alternative pathways to treatment, an insufficient research base inherently limits the utility of such orders. We then conclude with directions for future research.

## What is adolescent sexually abusive behaviour?

There is no universally agreed upon definition for ‘sexually abusive behaviour’ in young people (El-Murr, 2017; O’Brien, 2008), and nowhere is it defined in Victorian legislation (Judicial College of Victoria, 2019). Broader international literature indicates that although sexual experimentation is common throughout adolescence, sexual behaviours are considered abusive when they entail coercion, violence or a lack of consent (Hackett, 2010; O’Brien, 2008). It has also been argued that such behaviours should be classified as abusive only if they bring harm to a non-consenting victim, either directly or indirectly (McCuish & Lussier, 2017), or to the young person themselves (Allen, 2023). In their conceptual model of child sexual abuse, however, Mathews and Collin-Vézina (2019) argue that the infliction of harm is not a necessary condition; rather, exploitation of the victim and inequality within the victim–perpetrator relationship are more central to characterising abusive behaviours. Although this model does not necessarily pertain to sexually abusive behaviour specifically, this literature collectively highlights the lack of homogeneity in existing research when attempting to clearly define sexually abusive behaviour engaged in by young people.

Australian guidelines outline two key categories for harmful sexual behaviour in young people (see El-Murr, 2017). The first is problem sexual behaviour, which applies to children who do not meet the age of criminal responsibility (currently 10 years of age in Australia^1^) but are engaging in developmentally inappropriate sexual activities. For children aged 10 years and over (i.e. who do meet the legal age of criminal responsibility), this is referred to as sexually abusive behaviour. Although this dichotomy has been criticised due to being arbitrary (O'Brien, 2010; Royal Commission, 2017), the age of the young person is a key consideration as it helps to determine whether their behaviour deviates significantly from normative sexual development (Pratt et al., 2012, but also see Table 1). It also highlights the need to consider that some young people may engage in sexual behaviour that seems objectively abusive but may not be liable to a formal criminal justice response. The focus of this paper is on those young people who have reached the age of criminal responsibility in Australia and may be eligible for a formal criminal justice response for their sexually abusive behaviour.

**Table 1.** Examples of developmentally appropriate and inappropriate sexual behaviours in young people based on age.

Age range	Age-appropriate	Concerning	Very concerning
9–12 years	Masturbating in private ‘Show me yours, I’ll show you mine’ with peers Kissing and flirting with peers Talking about genitals or reproduction with peers Dirty words/jokes with peers	Attempting to expose others’ genitals Sexual knowledge too great given context Preoccupation with masturbation Peeping, exposing or seeking out pornography Simulating foreplay/intercourse with peers with clothes on	Compulsive masturbation Repeated/chronic peeping or exposing Chronic interest in pornography Forcing exposure of others’ genitals Penetration of dolls, other children, or animals Touching others’ genitals without permission
13–18 years	Sexually explicit conversations with peers Obscenities/jokes relevant to own culture Sexual teasing and flirting Masturbating in private Sexual intercourse plus full range of sexual activity with peers	Preoccupied or anxious about sex Seeking out pornography Being promiscuous Themes or obscenities involving sexual aggression Engaging in unsafe sexual behaviour	Compulsive masturbation Degradation of self/others with sexual themes Preoccupation with aggressive pornography Sexual contact with younger people or animals Sexual harassment

Note: Adapted from South Eastern Centre Against Sexual Assault and Family Violence (2017).

## An overview of young people who engage in sexually abusive behaviour

### Key characteristics

Young people who engage in sexually abusive behaviour are a heterogenous population, not restricted to any specific developmental antecedents, individual characteristics or offending patterns (McCuish et al., 2015). As such, a wide range of factors may be associated with the onset of sexually abusive behaviour, and few have received consistent empirical support. An eminent meta-analysis by Seto and Lalumiere (2010) found that factors such as a history of sexual abuse victimisation, early exposure to pornography, social isolation, higher anxiety and atypical sexual interests indicate specific risk for sexually abusive behaviour compared to Y-NSO. The authors also reported that Y-SAB have a less entrenched antisocial pattern, including fewer past criminal offences and fewer antisocial peers. In contrast, using a large sample which contained both males and females, Fox (2017) found that Y-SAB had an earlier age of offence onset and more prior arrests for serious crimes than Y-NSO, suggesting higher levels of antisociality. Sigurdsson et al. (2010) also noted that Y-SAB were more likely to self-report having delinquent peers than young people who denied ever engaging in sexually abusive behaviour. Discrepant results between studies may be at least partially explainable by considering within-group heterogeneity of Y-SAB – namely, the specialist/generalist typology asserts that some Y-SAB will be characterised by sexual deviance and engage in sexual offences exclusively, while others may engage in sexually abusive behaviour as part of a broader pattern of antisocial behaviour (Nisbet et al., 2010; Pullman et al., 2014).

Young people who engage in sexually abusive behaviour also demonstrate versatile patterns of offending over time (Carpentier & Proulx, 2011; Fanniff et al., 2017; McCuish et al., 2016). In a recent longitudinal evaluation of male adolescents who had been adjudicated for sexual offences, Rasmussen (2022) found that 6.2% were rearrested or reconvicted for sexual offences that required sexual offender registration over an average follow-up period of 12 years. Furthermore, a meta-analysis by Caldwell (2016) found that, over an average follow-up of five years, Y-SAB had a sexual recidivism rate of 4.97% and a general recidivism rate of 39.4%. This broadly aligns with a more recent meta-analysis by Lussier et al. (2023), who identified a sexual recidivism rate of 8% and a general recidivism rate of 44% over approximately five years. Collectively, these findings highlight that Y-SAB are more likely to reoffend non-sexually than sexually (Calleja, 2022). There is, however, considerable heterogeneity within Y-SAB as to the extent of their reoffending. This includes adolescent-limited offending, whereby sexually abusive behaviour takes place exclusively in adolescence, and life-course persistent offending, whereby for some young people their sexually abusive behaviour escalates to adult sexual offending (Lussier & Blokland, 2014; Moffitt, 1993; Moffitt et al., 2002). These trajectories have also been characterised in relation to the chronicity of offending, with some young people undertaking a much higher rate of both general and sexual offending in adolescence and adulthood (e.g. Cale et al., 2016).

### Effective treatment responses

Studies have noted that specialised treatment for Y-SAB may reduce both sexual and general recidivism over and above that for comparison groups (Ter Beek et al., 2018; Worling et al., 2010). The Association for the Treatment of Sexual Abusers (ATSA; a leading international, multi-disciplinary society for sexual abuse prevention) Adolescent Practice Guidelines (ATSA, 2017) advocate for a systems-based approach to treatment, whereby the young person’s social and environmental factors are incorporated into the intervention, including their family. This is in keeping with research that has found beneficial effects of multisystemic therapy on reducing offending in Y-SAB (e.g. Letourneau et al., 2013). The ATSA Adolescent Practice Guidelines also contend that although young people should be held to account for their behaviours, this should be done in a developmentally appropriate way, such as by incorporating significant adults into treatment as models for growth. Importantly, however, the benefit of specialised treatments on offending outcomes for Y-SAB remains contentious and inconclusive (Calleja, 2022; Ter Beek et al., 2018).

In accordance with prevailing guidelines (ATSA Adolescent Practice Guidelines, ATSA, 2017), community programmes for Y-SAB have received some empirical support. For example, Silovsky et al. (2019) found that a community-based early intervention programme for Y-SAB and their caregivers both reduced the frequency of sexually abusive behaviour and improved parenting skills. The sample was not restricted to young people who had undergone diversion, however, and there was no control sample. In another study, Laing et al. (2014) evaluated a community treatment programme for Y-SAB in New South Wales (NSW), Australia. Compared to a control group, Y-SAB who completed treatment had lower overall reoffending rates, especially for violent offences. No differences were found for sexual recidivism, however. In Victoria, over 90% of Y-SAB who undergo treatment in the community may at least partially meet their treatment goals (Pratt, 2013).

Although evidence-based interventions are undoubtedly critical to the rehabilitation of Y-SAB, young people must reasonably have appropriate access to such interventions to benefit from them (Munetz & Griffin, 2006; Shlonsky et al., 2017). Unfortunately, however, many studies do not identify the discrete legal or clinical pathway through which Y-SAB have accessed treatment. This is important as the means by which treatment is obtained may have significant bearing on offending outcomes. This is now discussed.

## Pathways to treatment

### Treatment following a youth justice order

Both in Victoria, Australia and internationally, a prominent pathway to treatment for Y-SAB has been court-mandated intervention during the enactment of a youth justice order (i.e. formal youth justice adjudication). Youth justice orders can be community based or custodial, whereby conditions of the order, including participation in treatment, are undertaken either in the general community or in a youth detention (‘correctional’) facility, respectively. The ATSA Adolescent Practice Guidelines (ATSA, 2017) suggest that most Y-SAB can safely engage in treatment in the community while custodial options should be reserved for those with the highest risk profiles. This aligns with the Risk–Need–Responsivity model (Bonta & Andrews, 2017), as well as findings that suggest that young people who receive treatment in detention facilities are likely to have engaged in more serious offending and have a higher risk profile than youth who are treated in the community (McCuish et al., 2015; Nisbet et al., 2010). Mandating treatment may also help address some of the risk factors associated with reoffending in Y-SAB, including treatment drop-out and reduced length of time spent in treatment, discontinuity of care or poor service coordination, and denial or minimisation of offending (Broner et al., 2005; Hunter & Figueredo, 1999; Lab et al., 1993).

Although custodial dispositions are legally considered to be a last resort for young people (Indig et al., 2016), The Australian Royal Commission into Institutional Responses to Child Sexual Abuse (2017; hereafter the Royal Commission) noted that in Australia, young people were more likely to be incarcerated for sexual offences than any other offence. It is therefore crucial to consider the treatment opportunities available for Y-SAB when they are formally adjudicated for their offending. In Victoria, Australia, the Male Adolescent Program for Positive Sexuality (MAPPS) was implemented in 1993 in response to growing acknowledgement that some sexual conduct engaged in by young people was inherently more harmful (i.e. abusive) than simple experimentation (Eger & Kilby, 1998; Flanagan, 2003). Young people can be mandated to participate in MAPPS via either a community or a custodial Youth Justice Order of the Children’s Court of Victoria (El-Murr, 2017), whereby they may engage in either group or individual treatment. Participation in MAPPS therefore occurs through a mandatory, adjudicative treatment pathway.

Despite being a staple of many correctional services internationally, mandating young people to treatment through formal adjudication processes poses a set of crucial challenges. In the Victorian context specifically, there was difficulty historically with successfully prosecuting Y-SAB aged from 10 to 13 years due to the prevailing Australian legal principle of *doli incapax* (from the Latin ‘incapable of deceit’), whereby a young person aged 10 to 13 years accused of a crime must be proven to have the capacity for criminal responsibility (Brown, 2019; Shlonsky et al., 2017). Although this principle serves an important protective function by accounting for necessary developmental considerations, such as the differing maturity levels of young people compared to adults (Craig, 2020), it made the successful prosecution of sexual offences perpetrated by young people difficult to achieve, and thus with no way to access treatment via MAPPS (O'Brien, 2010; Victorian Law Reform Commission, 2004). This concern is also broadly supported in international research – for example, in an analysis of federal data in the United States, Finkelhor et al. (2009) found that Y-SAB aged below 12 years had lower arrest rates (16.5%) than Y-SAB aged 12 years or older (32.9%). Without viable alternative pathways, therefore, treatment may be largely inaccessible to young people in these age brackets. This in turn may prevent opportunities for implementing early intervention (discussed further in ‘Diversion for young people with offending behaviours’).

If a young person is subject to a detention order, incarceration may have iatrogenic effects on young people more broadly. For example, it may interrupt pivotal developmental processes, especially in relation to social factors such as schooling, impede healthy attachment with a young person’s family and place young people at a higher risk of further trauma and victimisation (Gase et al., 2016; Royal Commission, 2017; Terry & Abrams, 2017; Worling & Langton, 2012; Zimring, 2000). In turn, this may mitigate the positive effects of any treatment the young person receives within the custodial environment (Gatti et al., 2009; Letourneau & Miner, 2005). For these reasons, it is important to consider alternative pathways to treatment than incarceration and mandated treatment.

### Diversion for young people with offending behaviours

Diversion is a broad concept, which can include both informal and formal mechanisms. Informal diversion includes circumstances such as police choosing not to charge a young person based on discretionary reasons, typically referred to as ‘cautioning’ (Munetz & Griffin, 2006; Shirley, 2017). In contrast, formal diversion can be broadly characterised as a legal process whereby people with unique behavioural, developmental or health needs are given access to community-based treatments as opposed to enduring a standard criminal justice response, thus avoiding possible prosecution and incarceration (DeMatteo et al., 2013; McAlinden, 2005). In line with this, diversion programmes vary according to which level of the criminal justice response they occur, and in general can be described as occurring pre or post official court action (Munetz & Griffin, 2006; Wilson & Hoge, 2013). Wilson and Hoge (2013) note that the true aim of diversion should be to limit a young person’s involvement with the criminal justice system as much as possible, emphasising the need to divert them before official legal action is taken.

Diversion for young people as an alternative to formal processing is internationally recognised as a fundamental principle when developing appropriate justice responses (United Nations General Assembly, 1985). Within the youth justice sector diversion comes in many forms (Campbell & Retzlaff, 2000), and though not all entail therapeutic intervention, there may be enhanced outcomes for young people in those programmes that include such interventions (Daly et al., 2013; Schwalbe et al., 2012; Seroczynski et al., 2016). Diversion also serves a protective function by keeping young people from the negative influence of the criminal justice system (Bouchard & Wong, 2017; Zimring, 2000). For example, Pope and Jones (2020) contend that a key benefit of diversion to community treatment is that developed skills can be utilised immediately, providing more of an incentive for behaviour change than treatment obtained while incarcerated. Importantly, estimates suggest that between 50% and 70% of young people successfully complete diversion (Campbell & Retzlaff, 2000; Loeb et al., 2015), and programme completion has been associated with lower reoffending rates in some studies (Laing et al., 2014; McGarrell & Hipple, 2007; Wilson & Hoge, 2013).

Another key benefit of diversion for young people is that it may help to facilitate early prevention and intervention. Homel et al. (2015) emphasise that early prevention is essential on a systemic level to stop offending from emerging or else becoming entrenched. This broadly aligns with commentary on sexually abusive behaviour specifically, in that many sexually abusive behaviours may have initially seemed innocuous or ‘normal’ only to have then escalated in severity (Anderson & Parkinson, 2018). In regard to offending generally, recent research has found that younger age of offending onset is associated with the persistence of offending into and across adulthood (Reil et al., 2021; Souverein et al., 2019). Diversion programmes for both sexual and non-sexual offending behaviours are often targeted toward young people who have not acquired extensive criminal histories (Allard et al., 2010; Bouchard & Wong, 2017; Campbell & Lerew, 2002), indicating that they enable early access to treatment or other diversionary mechanisms not only before offending behaviours increase in chronicity, but before young people become deeply embedded within the criminal justice system. Collectively, these considerations highlight that diversion pathways that enable young people to access treatment as early as possible are essential for any type of offence, including sexual offences.

Diversion programmes have also been broadly critiqued in international literature, however. For example, they have been argued to facilitate net-widening, whereby they bring a larger amount of young people into contact with criminal justice processes than otherwise would have occurred (Campbell & Retzlaff, 2000). Additionally, reductions in reoffending may be attributable to the young people being less surveyed by the justice system than those youth who undergo adjudication (Wilson & Hoge, 2013). In contrast, others have argued that undertaking a community diversion programme may mean the individual is more closely monitored and that further offending behaviour is more likely to be detected (Clarke et al., 2017; Schneider, 2010).

Perhaps the most important critique of diversion for young people is that empirical research has thus far been inconclusive regarding its effectiveness. Two meta-analyses found discrepant results, with Schwalbe et al. (2012) finding that diversion did not have a significant impact on recidivism while Wilson and Hoge (2013) concluded that young people who were diverted had lower reoffending rates than those who were on probation. This discrepancy has been attributed to the fact Schwalbe et al. utilised diversion programmes within their control sample, neutralising true effects, and because of the smaller number of studies identified by these authors (Wilson & Hoge, 2013; Wong et al., 2016). In another meta-analysis, Wong et al. (2016) found that diversion programmes based on principles of restorative justice effectively reduced recidivism in young people, but that there was considerable heterogeneity amongst studies. In particular, studies that included methodologically rigorous designs were less likely to show beneficial effects on recidivism. Collectively, these results indicate that the effectiveness of diversion programmes for young people has been inconclusive, and that rates of recidivism may be in part driven by methodological design.

There are also important cultural considerations to note. Mears et al. (2016) highlight that diversion pathways may risk exacerbating systemic biases within the criminal justice system, particularly against minority groups. For example, research findings demonstrate that Indigenous youth in Australia may be less likely to be diverted than non-Indigenous youth, even when both groups are first-time offenders and other offence-related variables are controlled for (Allard et al., 2010; Papalia et al., 2019). Furthermore, Wong et al.’s (2016) meta-analysis found that restorative diversion programmes delivered to predominantly ethnic minority youth did not significantly impact reoffending rates. These findings highlight that those young people who tend to be disproportionately impacted by involvement with the criminal justice system may be deriving the least benefit from important diversion pathways. Thus, although evidence for the efficacy of diversion is disparate and contentious, it is especially inconclusive for cultural minority youth.

In summary, although regularly endorsed as a viable treatment pathway for young people, diversion has so far received conflicting support in the literature. Benefits of diversion include that it protects young people from iatrogenic impacts of the criminal justice system, and also arguably serves as a form of early intervention. In contrast, diversion has also been argued to facilitate net-widening, and thus far limited evidence exists that supports the efficacy of diversion pathways for young people. Further research on diversion for youth is required.

### Diversion for young people who engage in sexually abusive behaviour

Internationally, many diversion policies have been legislated to engage Y-SAB in diversionary, rehabilitative programmes. It is beyond the scope of this article to describe these in extensive detail. A snapshot, however, is provided for common pathways encountered in the literature. For one, in South Africa, the *Child Justice Act (75 of 2008)* (South Africa) was passed and allows for the diversion of young people under a certain set of conditions, including that they take responsibility for their crime and that their parents or guardians consent to the diversion process (Abdulla & Goliath, 2015; Steyn, 2012). Various legal orders are a possibility, including a peer association order or a supervision and guidance order, with a referral agent (e.g. prosecutor) determining the most appropriate programme for the specific young person (*The Child Justice Act (75 of 2008)* (SA); Steyn, 2012). Furthermore, the United Kingdom has introduced Youth Offending Teams (otherwise referred to as Youth Offending Services), which respond to a young person’s offending as well as their unique needs (Haines et al., 2013; Smith et al., 2014; Youth Justice Board, 2013). Although these teams actively work with Y-SAB (Russell & Harvey, 2016; Smith et al., 2014), the effectiveness of this particular model has not, to our knowledge, been formally evaluated.

Various Australian and Oceanic locations have also introduced diversionary options for Y-SAB. For example, South Australia, South-East Queensland and New Zealand offer restorative justice conferencing options for Y-SAB as an alternative to a standard criminal justice process (Blackley & Bartels, 2018; Daly et al., 2013; O'Brien, 2010). Unfortunately, however, restorative conferencing options do not necessarily entail receiving specialised treatment for sexually abusive behaviour, and thus may not act as diversionary pathways to treatment. Although NSW offers community treatment to Y-SAB who have not been formally adjudicated (Laing et al., 2014), referrals are received from an array of sources and do not appear to necessarily be mandated (O'Brien, 2010). It is therefore unclear whether this acts as an official diversion pathway.

Several factors likely contribute to whether Y-SAB will be able to access diversion. Some of these accord with adult offenders, such as the severity of the index offence, any history of offending and their overall risk profile (Bouchard & Wong, 2017; Campbell & Lerew, 2002; Daly, 2006; Daly et al., 2013; Goodman-Delahunty & O’Brien, 2014; Loeb et al., 2015; McAlinden, 2005). Other factors include whether the young person had an extra-familial relationship with the victim, whether they were willing to admit to the offence, and their age (Daly, 2006; McAlinden, 2005; Mears et al., 2014). However, such factors may also relate to how likely the case is to be proven in court and thus to how much incentive there is for the young person to admit to the offence and undertake diversion (Daly, 2006; McAlinden, 2005).

Overall, there is limited research on the efficacy of diversion for Y-SAB. Some have endeavoured to characterise which young people undergo diversion (Campbell & Lerew, 2002), evaluate specific interventions embedded within diversion programmes for Y-SAB (Draper et al., 2013; Errington et al., 2013; Gxubane, 2016) or outline necessary practices for clinicians in the area (Gxubane, 2019). However, none of these studies addressed reoffending rates or other important criminogenic outcomes of the diversion pathway. Additionally, although evaluating the efficacy of specific treatments is informative, it does not allow for conclusions to be drawn about the pathway to treatment in and of itself (i.e. the benefits of diversion compared to adjudication; Daly et al., 2013). Conclusions related to the effectiveness of diversion to treatment for sexually abusive behaviour can therefore arguably not be drawn from these investigations.

There is, however, some limited work comparing outcomes related to pathways to treatment. Daly and Wade (2016) compared Y-SAB who underwent a diversionary conferencing programme to those who underwent a trial. Unfortunately, however, the authors focused exclusively on the experience of the victims. In another study, Daly et al. (2013) noted that Y-SAB with no prior offending who undertook a diversionary conferencing programme had longer time to general reoffending than those who underwent a court trial. Not all young people who undertook this diversion pathway accessed treatment, however, with only 56% referred to a specialised treatment service. Although accessing treatment was also associated with a longer time to reoffending than for those who were not referred to treatment, the authors were unable to analyse the combined effects of treatment access and placement in diversionary conferencing versus a court trial. Thus, when they examined the efficacy of specialised treatment, they did so by treating diversion and adjudication pathways to treatment homogenously. Based on the work conducted thus far, therefore, it is very difficult to identify which pathway to treatment is most beneficial for Y-SAB.

## Treatment pathways in Victoria, Australia

### A brief history

From the late 1990s to early 2000s, the situation in Victoria regarding pathways to treatment for Y-SAB was highly precarious. There was a greater focus on pursuing a criminal justice response over community intervention, and few Y-SAB would access specialised intervention unless successfully convicted and court-ordered to participate in MAPPS (Rodda, 2020). Additionally, the adjudication pathway was restricted primarily to those aged above 14 years (i.e. the age at which the legal principle of *doli incapax* no longer applies, thus making it more likely young people would be found criminally responsible for offences related to sexually abusive behaviour). This left many young people and their families without appropriate access to treatment or support, constituting a significant gap in the youth forensic treatment system in Victoria.

Prior to 2007, the only viable alternative to undertaking a criminal justice response was an informal diversion pathway whereby Y-SAB could access voluntary treatment in the community (Pratt, 2013). Various concerns also existed for this voluntary pathway, however. For one, many families were unable or unwilling to provide the necessary support structures to ensure young people complied with voluntary intervention (Pratt, 2013). This included living away from the home due to experiencing family violence (Royal Commission, 2017), leaving a significant number of Y-SAB with no way to successfully access treatment. Furthermore, in instances of intrafamilial sexually abusive behaviour, families were often forced to determine which child’s needs to prioritise and thus were reluctant to pursue community treatment for the child who had engaged in sexually abusive behaviour (Pratt, 2013; Rodda, 2020). Many authors have noted that familial support systems are necessary components for treatment and eventual reintegration into the community, and that ongoing familial dysfunction can risk compromising treatment success, including for Y-SAB (Gxubane, 2016; Laing et al., 2014; Steyn, 2012). Furthermore, with little prospect of criminal charges being pursued against them, many families and the young people themselves did not experience adequate incentive to engage with community services (Pratt, 2013). Thus, although this voluntary pathway was an alternative to adjudication, its utility was inherently limited.

**Figure 1.**
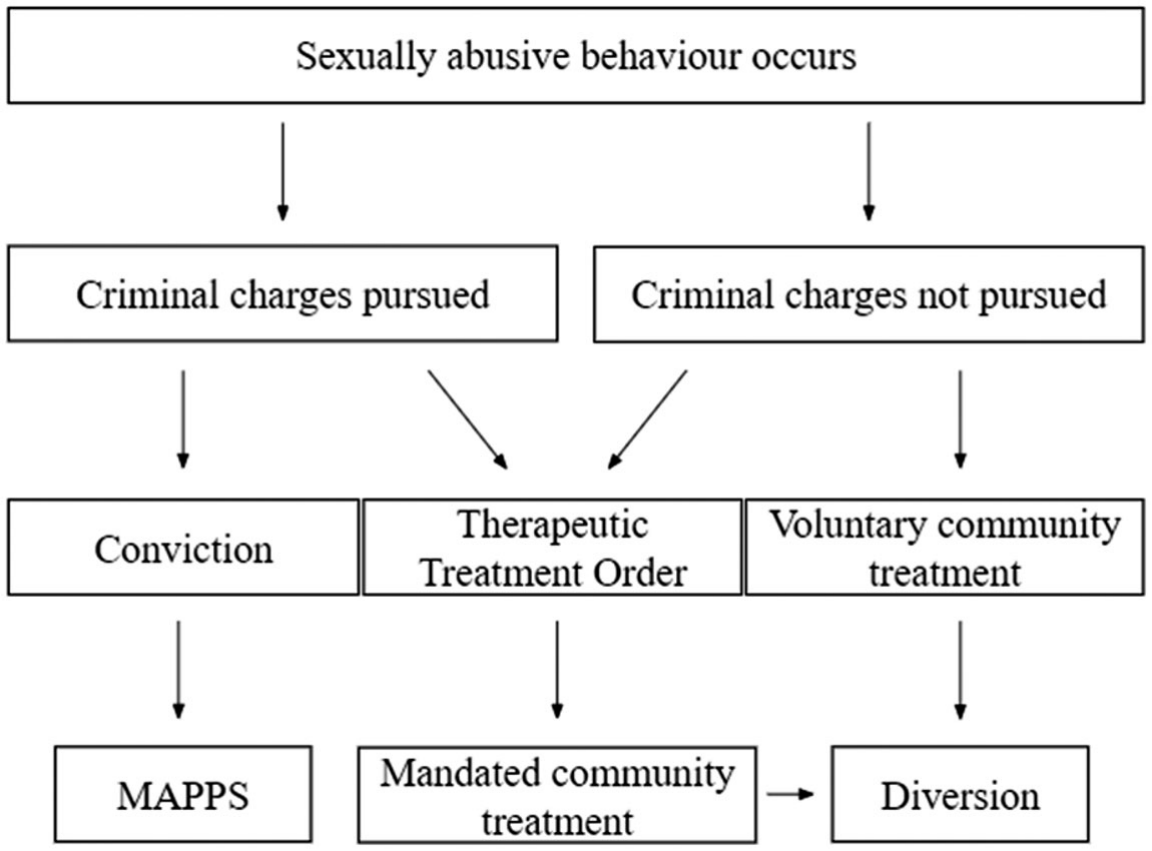
Pathways to treatment for sexually abusive behaviour in Victoria. MAPPS = Male Adolescent Program for Positive Sexuality.

After a comprehensive review highlighted the aforementioned issues (Victorian Law Reform Commission, 2004), the Victorian government enacted the *CYFA 2005* (Vic). This formally legislated not only voluntary community-based sexually abusive behaviour intervention, but also involuntary TTOs as a way to increase accessibility to treatment for Y-SAB. This extended the previous treatment pathways to a tripartite ‘Victorian model’ (Royal Commission, 2017, p. 120) for sexually abusive behaviour intervention, with Y-SAB able to gain access to treatment via three primary means: an involuntary pathway via a formal criminal justice response (i.e. adjudication), whereby the young person is ordered to attend MAPPS; a voluntary pathway to community treatment (e.g. via self-referral to a community service provider); and an involuntary pathway to community treatment via the provision of a TTO. See Figure 1 for a brief example of this service system.

### The introduction of Therapeutic Treatment Orders

**Figure 2.**
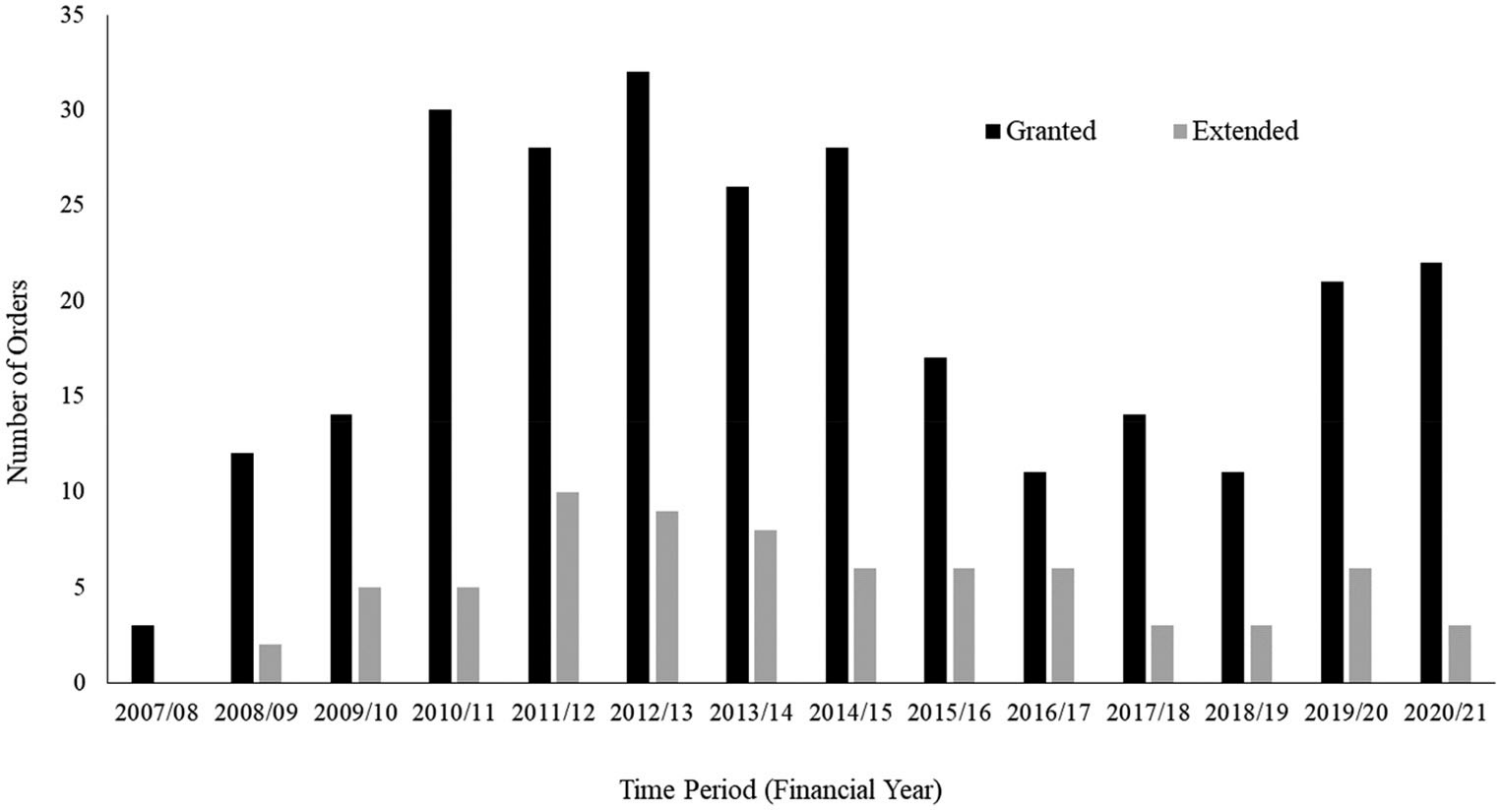
The number of Therapeutic Treatment Orders granted and extended. Note: Information in this figure was sourced from the Children’s Court of Victoria (2021).

A TTO is an order of the Family Division of the Children’s Court of Victoria, which mandates young people, initially aged between 10 and 14 years and then expanded up to 18 years, to attend community treatment when the court is satisfied they engaged in sexually abusive behaviour but may not be amenable to voluntary treatment compliance (Pratt, 2013; Rodda, 2020). Successful completion of treatment then results in further criminal charges not being pursued (*CYFA 2005* (Vic) s. 354), effectively diverting the young person from a formal criminal justice response (El-Murr, 2017). Shlonsky et al. (2017) consider this to be the key benefit of Victoria’s legislation, as the authors believe it incentivises the acknowledgment and treatment of sexually abusive behaviour. A recent legislative amendment also provides the same protections to young people undergoing community treatment voluntarily (*CYFA 2005* (Vic) s. 354 A), effectively creating both a mandatory and voluntary diversion pathway in Victoria. According to the Children’s Court Annual Reports (Children’s Court of Victoria, 2021), 269 young people have been placed on a TTO since they were first enacted in 2007 (see Figure 2).

**Figure 3.**
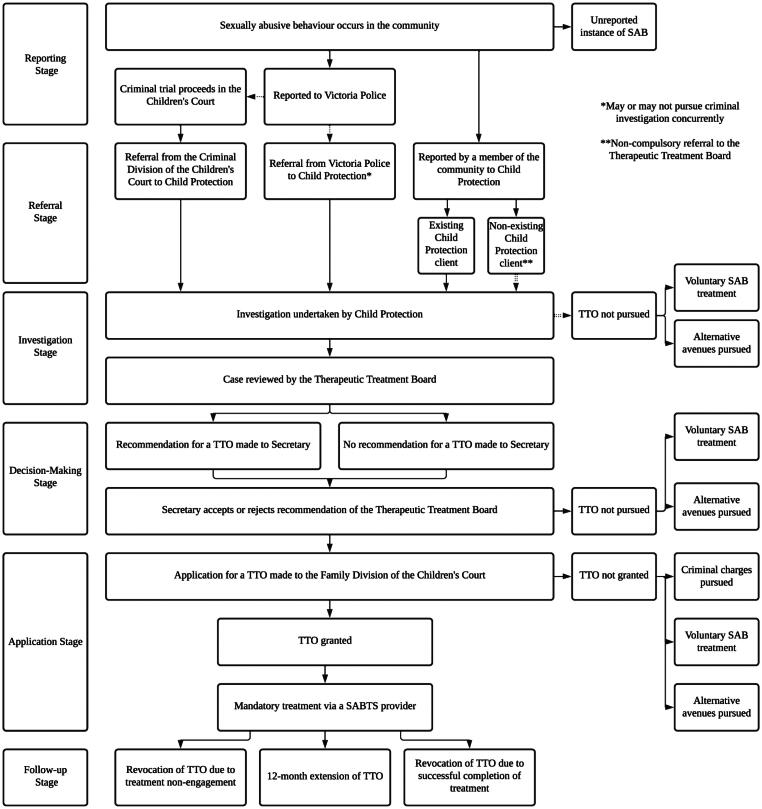
The pathway to obtaining a Therapeutic Treatment Order. Note: It is mandatory to refer young people to the Therapeutic Treatment Board unless they are a non-existing Child Protection client who has been reported by a member of the community. SAB = sexually abusive behaviour; SABTS = sexually abusive behaviour treatment service; TTO = Therapeutic Treatment Order.

An overview of the TTO model, including the referral process, can be found in Figure 3. Oversight for the orders is provided by two key bodies: the Victorian Child Protection Service (VCPS), embedded within the Department of Fairness, Families, and Housing (DFFH), and the Family Division of the Children’s Court of Victoria. The VCPS is responsible for investigating the alleged instance of sexually abusive behaviour. In the majority of cases, the VCPS (represented by the Secretary of the DFFH) is mandated to seek advice from the Therapeutic Treatment Board (TTB) as to whether a TTO should or should not be applied for (*CYFA 2005* (Vic) p. 4.13). The TTB comprises 16 stakeholders from a range of relevant disciplines, including police, prosecution and health services, who advise the Secretary whether a TTO is or is not appropriate for the particular young person (*CYFA 2005* (Vic) s. 245.6). The Secretary is then empowered to accept or reject this advice before applying for the TTO to the Family Division of the Children’s Court (*CYFA 2005* (Vic) s. 210, s. 515).

Although the VCPS tracks the young person’s treatment progress throughout the duration of the TTO, the Family Division of the Children’s Court has ultimate oversight of the order. Recent legislative changes expanded the Court’s ability to review the young person’s engagement with therapeutic intervention (*CYFA 2005* (Vic) s. 352 A). Furthermore, if the Secretary believes the young person is not engaging in treatment to a sufficient degree, they may apply for the TTO to be revoked, and criminal charges may be pursued in those instances where a trial has been adjourned (*CYFA 2005* (Vic) s. 258). Conversely, if the young person requires ongoing intervention, the TTO can be extended for a total of 12 months (*CYFA 2005* (Vic) s. 256).

### The need to evaluate Therapeutic Treatment Orders

While the adjudication pathway and the voluntary pathway in Victoria arguably represent traditional approaches to placing Y-SAB in treatment, the TTO pathway effectively acts as a bridge which incentivises young people and their families to access treatment in the community without having to endure stigmatising and stressful criminal justice processes. As such, TTOs arguably overcome many of the limitations of traditional pathways to treatment. For example, given that some young people placed on TTOs are identified at the child protection level (O’Brien, 2010), this introduces the possibility of addressing sexually abusive behaviour before the young person ever faces formal adjudication. TTOs therefore adhere to tenets of early intervention by addressing sexually abusive behaviour before the behaviour, or its resultant consequences, escalate in severity. This is especially true in comparison to alternative treatment models (e.g. *Child Justice Act (75 of 2008)* South Africa; Daly et al., 2013; Laing et al., 2014) where admission of guilt is required to access diversion, potentially limiting the amount of young people for whom early intervention can be facilitated. For TTOs, it is only necessary that *prima facie* evidence exists (*CYFA 2005* (Vic) s. 349.3) – that is, that it is very likely the sexually abusive behaviour occurred, considering matters such as the severity of the sexually abusive behaviour and any prior history of sexually abusive behaviour. These orders are therefore arguably more accessible than alternative forms of diversion.

There are other key benefits of TTOs. For one, in line with prevailing treatment guidelines (ATSA Adolescent Practice Guidelines, 2017), young people are treated in the community with a focus on the systemic drivers of offending. It may therefore allow young people to utilise learnt skills right away (Pope & Jones, 2020) and to have their families or other social supports incorporated into treatment in a meaningful way. This is also a testament to how TTOs address limitations of receiving treatment through adjudication, particularly a custodial sentence, including isolation from support networks, adoption of a deviant identity and the stigma associated with incarceration (United Nations General Assembly, 1985; Wong et al., 2016). Furthermore, TTOs may also arguably motivate, or help to incentivise, treatment compliance (Shlonsky et al., 2017). As Pratt (2013) notes, faced with the prospect of adjudication should treatment non-engagement occur, many young people and their families may be more likely to comply with the conditions of the TTO and engage with the community service provider. Finally, in other jurisdictions, Y-SAB may be restricted from accessing community treatment due to agency disagreement on the most appropriate management framework for the young person (Laing et al., 2014). TTOs resolve this by standardising the referral process and having a body of experts (i.e. the TTB) advising on the case.

It is important to note that TTOs may also have limitations. Prospects for treatment success may be increased when it is accessed voluntarily (Lösel & Schmucker, 2005; McAlinden, 2006; Smallbone et al., 2009), and mandatory treatment may be associated with reduced intrinsic motivation and more resistance to care, and thus a higher possibility of reoffending (Birgden, 2007; Levenson & Prescott, 2009). Unfortunately, however, many studies do not specifically outline the mechanisms through which those who have engaged in sexual offences enter treatment – indeed, recent reviews have noted that around 25% of studies do not explicitly note whether sexual offender treatment is being accessed voluntarily or involuntarily (Lösel & Schmucker, 2005; Schmucker & Lösel, 2015). This is despite Y-SAB potentially being mandated to treatment more often than other youth offenders (van Wijk et al., 2007). Where this information is noted, it is generally not considered as a possible confound. Given that TTOs rely on mandatory treatment referral, it would be important to address whether the nature of involuntary treatment has adverse consequences on reoffending outcomes.

Other limitations may also be present. Given that TTOs entail young people who may not have otherwise faced court action for their sexually abusive behaviour, there is no doubt that an argument for net-widening could be made. The orders may also place young people under harsher surveillance, meaning that further sexual or non-sexual offending may be more likely to be identified and potentially risking further criminal processing (Clarke et al., 2017; Schneider, 2010). Furthermore, it is unclear the extent to which this treatment service model adheres to key risk, need and responsivity principles given that it has never been formally evaluated, and that treatment programmes across different community service providers vary in nature. Finally, there have been concerns raised in the literature that implementing diversion without sufficiently addressing familial dysfunction may not allow for treatment gains to be consolidated (Steyn, 2012). This is especially crucial for young people on TTOs given it was in part implemented for those young people who do not have sufficient familial support. Although community treatment providers in Victoria approach intervention from a trauma-informed and systemic lens (it is beyond the scope of this article to discuss treatment approaches for Y-SAB in Victoria in detail; see Pratt, 2013, 2014 for more information), the success of this approach for young people on TTOs is unclear. These limitations do not mean that efforts to establish diversion pathways should be abandoned, nor that TTOs are inherently problematic. Rather, they reiterate the importance of ensuring such diversion pathways are rigorously evaluated to determine whether they are achieving their intended purpose – meeting the individual needs of young people while reducing the prospects of future offending.

## Conclusion and future directions

This article provided a critical overview of typical treatment pathways utilised for Y-SAB who engage in offending, including mandatory treatment via adjudication and treatment via diversion pathways. It has demonstrated that although diversion is an increasingly utilised legal provision for young people, benefits of this pathway remain largely within the theoretical realm, with a limited body of work supporting the claim that it is a more beneficial treatment pathway than traditional criminal processing (Mears et al., 2016). For Y-SAB in particular, limited work has been dedicated to determining differences in criminogenic outcomes between those who are diverted to treatment or adjudicated.

This article has also provided the first comprehensive overview of TTOs in the research literature. In Victoria, Australia, TTOs act as a mandatory form of diversion, which allow Y-SAB with complex familial backgrounds or poor treatment motivation to both access early intervention and avoid the potential iatrogenic effects of the criminal justice system. Although we have explored how TTOs may overcome many of the limitations associated with other treatment pathways (i.e. via adjudication or voluntary processes), we have also determined that TTOs may contain several shortcomings, perhaps the most crucial being that they have never been formally evaluated.

Given the ethical concerns associated with mandatory treatment and the adverse consequences of incarceration on young people, an evaluation of whether TTOs (and, arguably, other diversionary treatment pathways) can effectively address sexually abusive behaviour is crucial. This would not only contribute to a limited literature on the effects of diversion on outcomes for Y-SAB but would have important implications for increasing the application of mandatory treatment diversion programmes in youth justice both nation-wide and internationally. In particular, it would be important to characterise the individual characteristics and offence trajectories of Y-SAB undertaking each of the three pathways to treatment in Victoria, paying particular focus to whether the highest risk individuals are receiving the most intensive treatment (i.e. does the Victorian Model adhere to the Risk–Need–Responsivity Model?). Given the research discussed here, it would also be crucial to determine whether Y-SAB with specific characteristics (e.g. familial dysfunction, Indigenous youth) are likely to gain similar therapeutic benefits from each of the three pathways. A large part of this would be seeking to understand the experience and attitudes of young people who have undertaken different pathways to treatment, such as by interviewing them on issues related to their experience of mandated versus voluntary treatment.

Although TTOs in Victoria contribute much to international knowledge on how to implement diversion, we acknowledge that endorsing this scheme without empirical data contributes to a deeply entrenched issue. Across the previous two years we have made attempts to initiate a research project evaluating and comparing the efficacy of the different treatment pathways for Y-SAB in Victoria, especially TTOs. Due to the various concerns associated with research of this nature, however, including the highly sensitive nature of information, vulnerability of young people undergoing treatment and legislative limits in regard to the privacy and accessibility of data, we have not yet been successful. We do believe, however, that there is no better place to begin this empirical journey than by providing a concise overview of the treatment system for Y-SAB in Victoria. This will contextualise all further research undertaken and will hopefully serve to incentivise others internationally to provide clear, coherent accounts of their service systems.

## Footnotes

1 In Australia, criminal law (and thus criteria for the age of criminal responsibility) is legislated independently by each State or Territory government. Currently, there is growing national discussion and advocacy that the age of criminal responsibility should be raised to 14 years by each state.
